# Synthesis and Investigation of Cryogenic Mechanical Properties of Chopped-Glass-Fiber-Reinforced Polyisocyanurate Foam

**DOI:** 10.3390/ma14020446

**Published:** 2021-01-18

**Authors:** Jeong-Dae Kim, Jeong-Hyeon Kim, Dong-Ha Lee, Dong-Ju Yeom, Jae-Myung Lee

**Affiliations:** 1 Department of Naval Architecture and Ocean Engineering, Pusan National University, Busan 46241, Korea; jeongdae3416@pusan.ac.kr (J.-D.K.); honeybee@pusan.ac.kr (J.-H.K.); dongha2@pusan.ac.kr (D.-H.L.); ehdwnshdwkd@pusan.ac.kr (D.-J.Y.); 2 Hydrogen Ship Technology Center, Pusan National University, Busan 46241, Korea

**Keywords:** synthesis, mechanical properties, compression, thermal properties, reinforcement

## Abstract

Polyisocyanurate foam (PIF) has been adopted as a liquefied natural gas (LNG) insulating material owing to its various mechanical merits such as high structural stability and mechanical strength, and excellent insulating ability. In an attempt to increase the mechanical strength of PIF, chopped-glass-fiber-reinforced polyisocyanurate foam (CGR-PIF) was synthesized by adding chopped glass fibers to polyol and isocyanate, which are the raw materials used in the polymerization process for producing PIF. The main objective is to closely observe the compression material characteristics of PIF and CGR-PIF in terms of the cryogenic temperature. Therefore, compressive tests were conducted at cryogenic temperature including low temperatures, and microscopic images were obtained to analyze the cell size and distribution that affects the mechanical and thermal properties of the foam. Furthermore, recovery ratio and weight loss which are important factors of brittle fracture were evaluated, and the applicability of the foams to a cryogenic environment was evaluated. Finally, thermal conductivity, an important parameter of insulation, was evaluated. The obtained results confirm that the compressive strength of CGR-PIF significantly increases at cryogenic temperatures; moreover, a relatively higher thermal conductivity was observed in the case of CGR-PIF as compared to that of PIF owing to the chopped glass fibers.

## 1. Introduction

**Figure 1:**
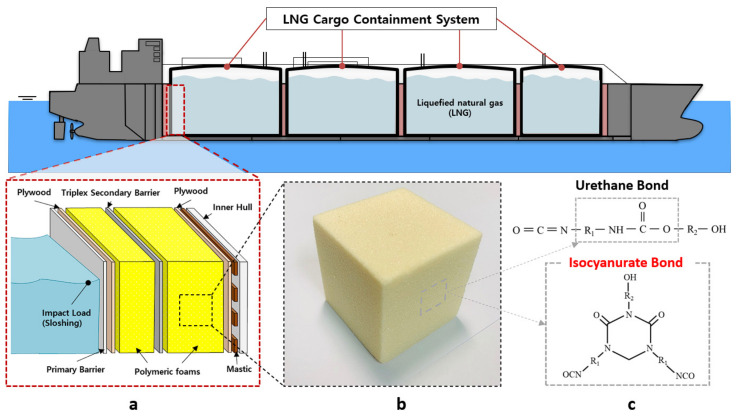
Schematic of the liquefied natural gas (LNG) insulation system. (**a**) cross-section of LNG insulation system (**b**) polymeric foam used in system (**c**) molecular structure of polyurethane foam (PUF) and polyisocyanurate foam (PIF).

The global energy demand is increasing owing to the growing population and expanding economies. Furthermore, natural gas is already an essential green energy source for fulfilling the global energy demand. The natural gas demand is expected to grow by 4.4% each year till 2030, which will make it the most popular fossil fuel [1]. The liquid form of natural gas at −163 °C at atmospheric pressure is called liquefied natural gas (LNG). It is the cleanest form of natural gas as it does not emit sulfur during combustion. As the demand for LNG has increased owing to concerns over environmental pollution, LNG insulation systems have been become the focus of attention in the clean energy industry [2,3]. LNG is contained and transported at a temperature of −163 °C in a liquid state in order to reduce its volume for the purpose of storage [4]. A temperature difference of approximately 190 °C is generated between the LNG storage tank and the outside of the tank, thereby resulting in a boil-off rate (BOR) due to the occurrence of heat transfer. BOR means the rate of natural vaporization and escape during the storage and transportation of LNG. The lower the rate of vaporization, the greater the original amount of LNG that can be conserved. In order to reduce the BOR, materials with excellent insulation performance are required. In addition, sufficient mechanical strength is required in LNG insulation systems because of various loads such as self-gravity, the dynamic load caused by liquid movement, and the pressure induced by liquid vaporization inside the system. For this reason, as shown in Figure 1, in LNG insulation systems, rigid polymeric foams have been selected as the core insulation material in the sandwich panel for reducing the heat transfer and various loads [5].

Polymeric foams are widely used as insulation materials in sandwich structures because of their low density, excellent energy absorption ability, low moisture permeability and good insulation performance [5,6]. Rigid polyurethane foam can be distinguished as polyurethane foam (PUF) and polyisocyanurate foam (PIF) among the organic and inorganic insulation materials, and they are employed as insulation in buildings, LNG gas storage tanks, and insulating materials in LNG pipes. Glass-fiber-reinforced polyurethane foam, named R-PUF, has primarily been adopted for Mark-III type LNG cargo containment systems in order to withstand the fluid impact load. Polymeric foams used in LNG containment systems are required to withstand not only the sloshing and self-gravity of LNG but also to minimize the BOR and protect the hull structure from the effects of cryogenic temperatures [7]. In particular, PIF, which is a thermal insulation material that can be used over a wide range of temperatures (−235 to 230 °C), has a lower thermal conductivity (0.02–0.03 W/mK) than other polymeric foams such as PUF (0.032 W/mK) and R-PUF, which are commonly used in membrane cargo tanks that are used to store LNG [8,9,10].

PIF has been developed to have superior thermal stability and flammability characteristics. PIF can be synthesized without the use of polyol but requires the use of isocyanate, a catalyst, a blowing agent, and a foam stabilizer (surfactant). Instead of reacting with the polyol, the isocyanate reacts with itself to form a highly cross-linked thermosetting neutralized body with a cyclic structure. Owing to this isocyanurate bonding as shown in Figure 1c, PIF has a low thermal conductivity, high fire retardancy, and high dimensional stability [8,11,12].

Owing to the excellent physical and mechanical properties of polymeric foam, several research studies have been conducted on the mechanical properties of PUF. The room-temperature mechanical properties of closed-cell polyurethane foam were measured at various values of density, which influences its mechanical performance [6,13]. Park et al. investigated the mechanical properties of various polymeric foams (PUF, R-PUF, and PIF) and evaluated the cryogenic-temperature material characteristics to reveal that PIF showed superior mechanical properties when the effect of density on its mechanical properties was minimized [8].

However, the problem of breakage of the polymeric foam used in the insulation system continues to occur, and studies are underway for enhancing the performance of polymeric foams. Generally, the method used for improving the performance of a polymer foam includes adding fibers and particulates to the foam. Zhao et al. analyzed the mechanical and thermal performances of granular silica aerogel reinforced PIF. It was found that the higher the silica aerogel ratio was, the lower its thermal conductivity was, and its compressive strength was slightly improved [14]. Nevertheless, embrittlement occurred when the reinforced PUF exposed to a cryogenic temperature owing to low cohesion with particulates [10,15]. To overcome this limitation, the effect of the use of glass fibers in PUF was also investigated, and it was revealed that R-PUF had a 20% higher compressive strength than that of PUF. In addition, glass fiber reinforcement increased the cohesion of PUF, which helped it to keep its original structure [16,17]. A compression test, 3 point bending test and tensile test were carried out varying the amount of glass fiber to be synthesized with polyurethane foam by 5%, 10%, 20%. As a result, it was found that the strength of the foam increased gradually until the amount of glass fiber reached 10%, but the strength of the foam did not increase further with an increase in the amount of glass fiber used [18].

Although research has been conducted on the performance of PUF with the addition of glass fibers, the synthesis of glass fibers for PIF, which has different molecular structure, and its mechanical performance evaluation at various temperatures has not been conducted. The majority of the studies on this subject are focused on the mechanical performance of the foam at room temperature or a low temperature and have not evaluated its mechanical performance at cryogenic temperatures, such as those used for storing LNG.

When a polymeric foam having a relatively high density is used, the weight of the hull is increased, thus resulting in economic loss. In order to overcome this insulation system problem, it is necessary to improve the mechanical strength per unit density and thermal performance of the polymeric foam. Although PIFs are widely used in insulation systems, a few studies have been conducted on the effects of glass-fiber reinforcement and no cryogenic mechanical performance evaluations have been performed when PIFs are reinforced with glass fiber [19].

In this present study, the main objective is to closely observe the compression material characteristics of PIF and chopped-glass-fiber-reinforced PIF (CGR-PIF) in terms of the cryogenic temperature. Therefore, compressive tests were conducted with PIF and CGR-PIF at various temperatures, and SEM micrographs were also obtained in order to analyze the cell shape and distribution that affects the mechanical and thermal properties of the foam. In addition, the recovery ratio and weight loss were evaluated, and the applicability of the foams to a cryogenic environment was evaluated. Finally, the thermal conductivity, as an important parameter of insulation, was evaluated.

## 2. Experimental

### 2.1. Materials

**Figure 2:**
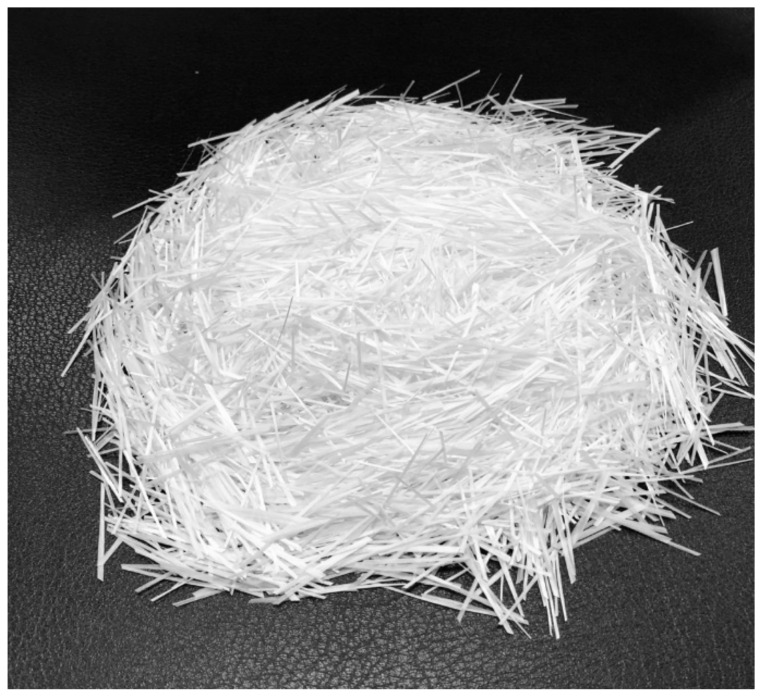
Photograph of chopped glass fibers.

The polyol mixture was used to prepare PIFs with commercially available polyols from BASF company (Ludwigshafen, Germany)—Lupraphen 1901/1 (difunctional polyester polyol based on aromatic dicarboxylic acids). Properties of the polyol mixture used in this study are summarized in Table 1. Isocyanate (Lupranat M50) used in the reaction was 4,4′-diphenylmethane diisocyanate (MDI) characterized by a 31.5% content of NCO groups. HFC 365–227 was used as a chemical and physical blowing agent. Chopped glass fibers (length: 25 mm) were purchased from FRP shop, Korea. Table 2 lists the material characteristics of the chopped glass fibers, and Figure 2 shows the chopped glass fibers used in this study.

### 2.2. Synthesis of the CGR-PIF

**Figure 3:**
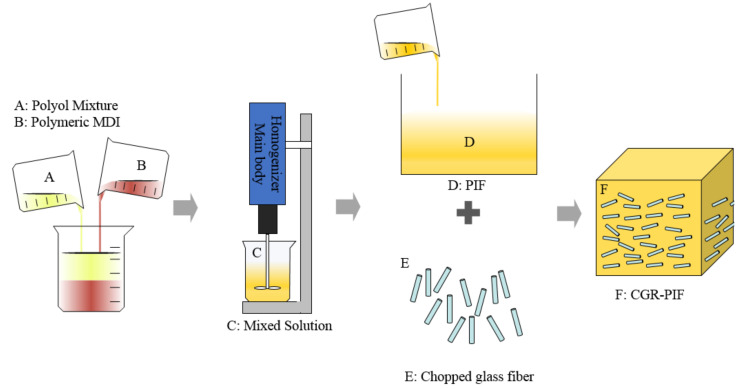
Manufacturing process of chopped-glass-fiber-reinforced polyisocyanurate foam (CGR-PIF).

PIF is a cellular, thermoset plastic formed when the two liquids polyol and M50 are combined. In this study, CGR-PIF is fabricated to investigate the effect of chopped glass fibers in PIF. The main constituents of the CGR-PIF are polyol, M50, and chopped glass fibers. Figure 3 illustrates the manufacturing process of the CGR-PIF. Polyol, M50, and the chopped glass fibers were combined in a mass ratio of 180:100:50 to prepare a 15 wt.% CGR-PIF because the R-PUF manufactured in previous studies with 10–15 wt.% of glass fibers had good mechanical properties [18]. The composition and mixing conditions of PIF and CGR-PIF are shown Table 3. Before adding the chopped glass fibers, polyol and M50 were mixed using a homogenizer (IKA, Staufen, Germany) for 15 s at 6000 rpm. The well-mixed PIF liquids were poured into molds (350 × 350 × 300 mm^3^) to produce free-rise foam. One half was poured in an empty mold to produce pure PIF. The other half of the mixture was poured into a mold with chopped glass fibers at its bottom. The liquids were then pressed to mix with the chopped glass fibers and to align the fibers in the horizontal direction. After 24 h at an ambient temperature of 20 °C, the polymerization reaction was completed.

### 2.3. Processing

**Figure 4:**
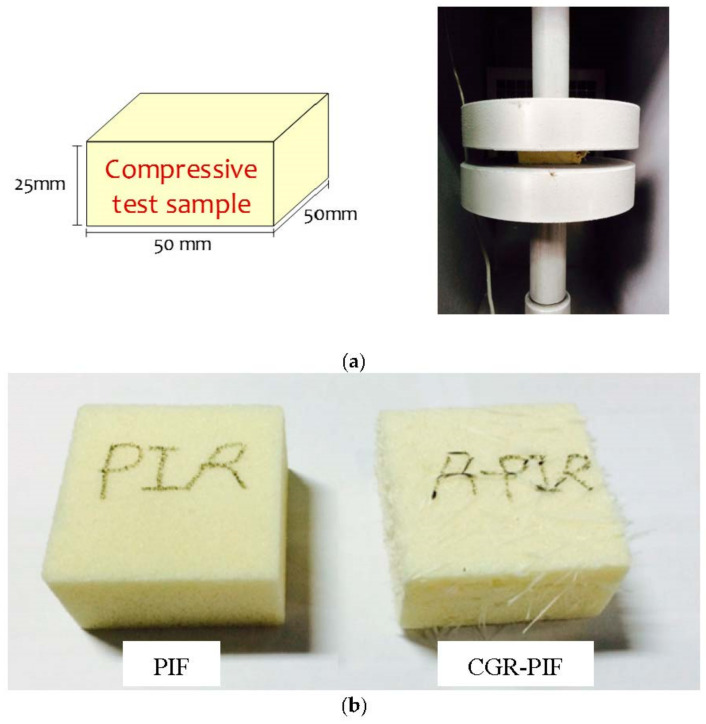
(**a**) Schematic of sample for compression tests and (**b**) machined specimens for compression test of PIF and CGR-PIF.

Figure 4a shows the schematic of the compression test specimen and compression test jig. The density of the PIF increased with the addition of chopped glass fibers. The PIF (mass density = 59 kg/m^3^) and CGR-PIF (mass density = 75 kg/m^3^) were subjected to compression tests and a brick-type specimen of size 50(B) × 50(W) × 25(H) mm^3^ was machined in accordance with the Korean Industrial Standard (KS M ISO844). In addition, compression test specimens were machined in the foam rising direction, which is the main direction in which the self-gravity of the LNG and the sloshing load act. Figure 4b shows the photograph of the machined specimen used for the compression test of PIF and CRG-PIF. The compression test specimens were arbitrarily selected, and the average value of their density was determined. As a result, it was confirmed that the density of the PIF was approximately 59 kg/m^3^ and that of the CGR-PIF was approximately 75 kg/m^3^. For the field emission SEM (FE-SEM) analysis, the X–Y plane (perpendicular to the foam rising direction) and Z-plane (parallel to the foam rising direction) of the PIF and CGR-PIF which is not conducted the compression test were sampled and analyzed. The test specimens for the thermal property analysis were fabricated with a size of 1(B) × 1(W) × 0.2(H) mm^3^. Five specimens were randomly selected in bulk, and a graphite coating was applied on them in order to enhance their light absorption.

### 2.4. Test Apparatus and Scenario

**Figure 5:**
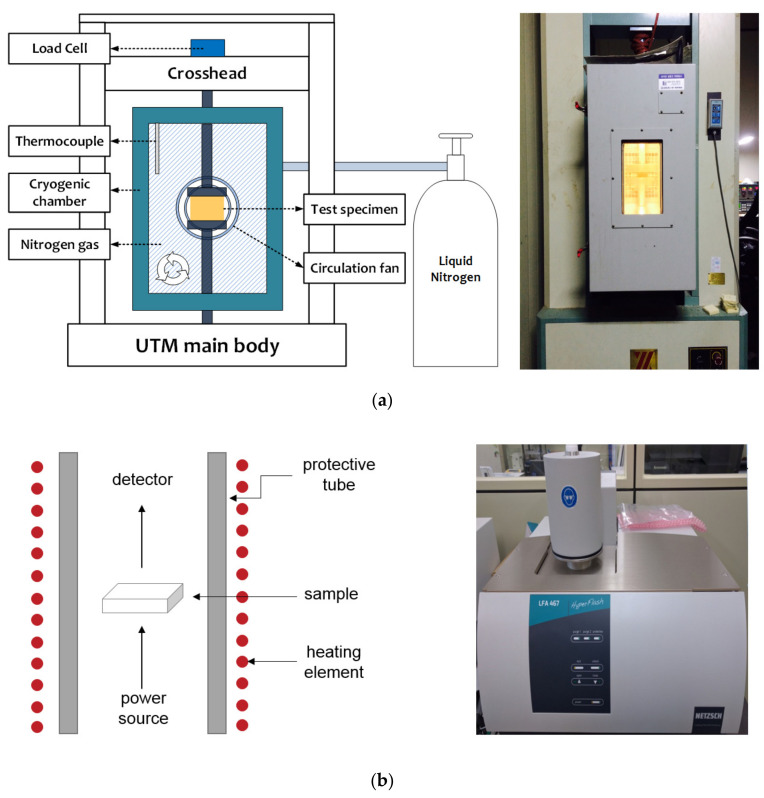
Photographs and schematic of test apparatus. (**a**) Universal testing machine equipped with cryogenic chamber. (**b**) Laser flash analysis.

In this study, a homogenizer was used to mix the polyol and M50 at 6000 rpm. As shown in Figure 5a, a universal testing machine (KYOUNGSUNG TESTING MACHINE CO., LTD, Ansan, Korea) for composite materials equipped with a cryogenic chamber and automatic temperature control system was used to investigate the compressive strength of the foams. When nitrogen gas was injected in order to lower the temperature to the cryogenic temperature, the automatic temperature control system prevented the further injection of nitrogen gas once the temperature reached the desired value, and when the temperature started to increase, it lowered the temperature through re-injection such that it was maintained at the desired value. The strain rate was set as 0.001/s (1.5 mm/min) in consideration of the quasi-static load. For the thermal equilibrium of the specimens, precooling was performed for 1 h, and then, compression tests were performed. Table 4 shows the conditions under which the compressive strength was evaluated. In order to analyze the cell morphology of the PIF and CGR-PIF, scanning electron microscopy (FE-SEM SUPRA25, ZEISS, Oberkochen, Germany) was used. In addition, the thermal conductivity was measured using laser flash analysis (LFA, Netzsch, Selb, Germany), which is widely used to measure the thermal diffusivity of metals, composites, ceramics, and other materials by using laser pulses in the temperature range of room temperature to 500 °C. The LFA comprised three parts: the light source at the bottom, the sample in the center, and the detector on top. A laser pulse from the power source hits the bottom of the sample. As the laser pulse energy was absorbed at the surface of the sample, the temperature of the rear surface was monitored using the detector. By measuring the thermal diffusivities of the specimen, the thermal properties such as thermal conductivity and specific heat were calculated [20].

## 3. Results and Discussion

### 3.1. Morphological Characterization

**Figure 6:**
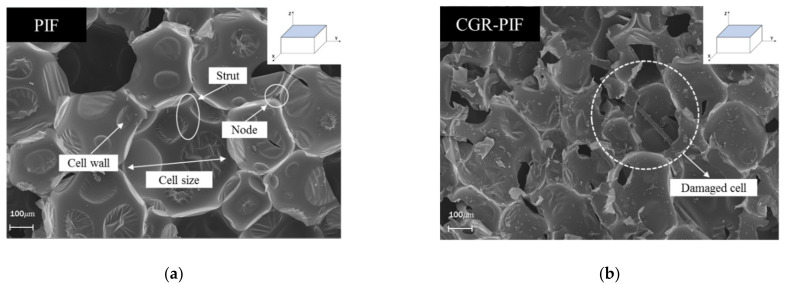
Cell morphologies of the PIF and CGR-PIF along the surface perpendicular to the foaming direction for (**a**) PIF and (**b**) CGR-PIF.

**Figure 7:**
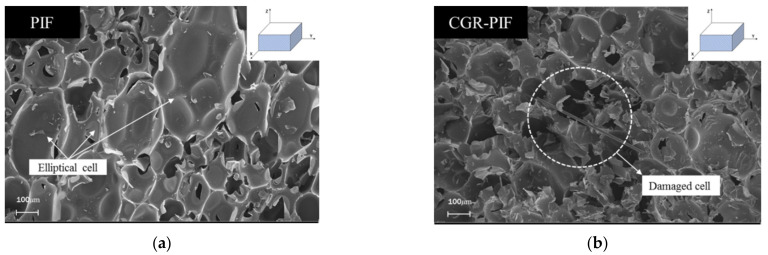
Cell morphologies of the PIF and CGR-PIF along the surface parallel to the foaming direction for (**a**) PIF and (**b**) CGR-PIF.

The rigidity of the polymeric matrix and the cellular structures of the foam influence the mechanical and thermal properties of the polymeric foam [21]. Polymeric foams can be classified as open-cell and closed-cell structure foams [22]. The closed-cell structure has a greater absolute thermal resistance (R-value), which reduces the heat flow, than that in the case of the open-cell structure, and thus, the closed-cell structure has a superior insulation capability. The PIF considered in this study had a closed-cell structure as observed in the SEM images. The effect of the chopped glass fibers on the cellular morphologies of the foam was examined using FE-SEM. The SEM image can be used to understand the structure of the cell, cell distribution, and whether the cell is destroyed. Figure 6 and Figure 7 show the FE-SEM morphology of the PIF and CGR-PIF cell structure surfaces perpendicular and parallel to the foaming direction. The cell size was measured by setting the length in the direction of the longest length while considering that the structure of the cell was anisotropic. Table 5 shows the mean, maximum size, and standard deviation of the cell size defined earlier. In the direction perpendicular to the foaming direction of the PIF and CGR-PIF, it was observed that the standard deviations—which indicate the uniformity of the cell structure—were similar, but it was confirmed that the cell size was larger in the case of PIF. This is because the addition of chopped glass fibers affected the form of the cell structures. It was observed that the cell growth around the glass fiber was hindered by the glass fiber, and consequently, the cell size around the chopped glass fiber was relatively small [23,24]. This is owing to the viscosity of the glass fiber and the PIF liquid resulting from the foaming process. The formation of small cells owing to the presence of the chopped glass fibers led to a high density of the foam, thus resulting in a higher mechanical strength [14,25]. The structure of the cell in the foaming direction, which is the same as the direction of heat transfer, self-gravity, and sloshing, affected the heat insulation performance and mechanical strength. In the case of PIF, it was confirmed that the degree of anisotropy was larger in the direction parallel to the foaming direction than in that perpendicular to the foaming direction. In addition, as the standard deviation was large, it could be observed that cells had a relatively large wide of cell sizes. In terms of cell size, it could be predicted that the PIF had a larger cell size on average than the CGR-PIF, and thus, its insulation performance was better. However, in terms of mechanical strength, it could be predicted that the compressive strength of CGR-PIF, which had a relatively uniform cell size and small average size, would be higher. Its mechanical properties were improved—in the form of a high compressive strength—by adding glass fibers. However, it is considered that its thermal conductivity was increased owing to the addition of glass fibers. In fact, the thermal conductivity of R-PUF reinforced with glass fibers has been reported to be higher [26].

### 3.2. Deformation Behaviors

**Figure 8:**
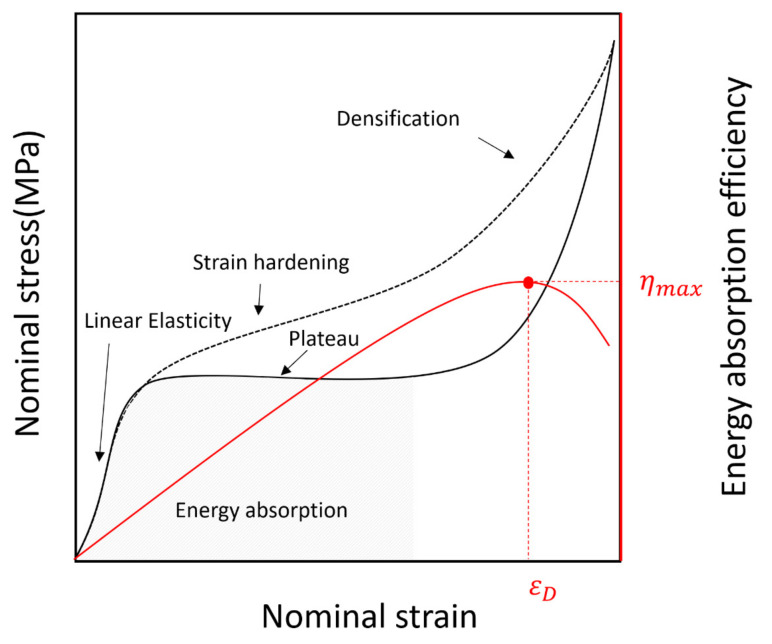
Typical compression stress–strain curves for polymeric foam.

Figure 8 shows the typical deformation mechanisms of a polymeric foam. When the polymeric foam was subjected to a compression load, the three regions of linear elasticity, a plateau, and densification were observed consecutively [27,28]. In the linear elastic region, the stress and strain were proportionally increased and returned to their original state when the applied force was removed. This was usually observed at a strain of 5% to 10%, and the slope of the stress–strain curve in the linear elastic region represents the modulus of elasticity (Young’s modulus). The long plateau region extended from the yield stress point where the linear elastic region ended, and the stress was almost constant even though the strain increased in this region [29]. Furthermore, depending on the polymeric cell structure, strain hardening occurred wherein the stress gradually increased before the occurrence of a fracture. Generally, in the case of low-density polymeric foam such as PIF with a relatively non-uniform cell size, when a compression load is applied, the larger sized cells collapse before the smaller ones. However, when a polymeric foam having a high density, small cell size, and uniform cell structure is compressed, the stress resistance to deformation beyond the elastic region is increased as compared with that in a low-density polymeric foam. This causes the stress to increase until the cell is completely collapsed [30]. As the opposing cell walls approach each other and meet, the stress increases steeply owing to the compression effect of the residue that escapes the internal blowing agent gas.

When a load is applied to the polymeric foam, work is done by the forces acting on it, which is called specific energy. The work per unit volume (specific energy) used to deform it to a densification strain is represented by the area under the stress–strain curve up to $\varepsilon_{D}$. The densification strain, $\varepsilon_{D}$, is the strain corresponding to the maximum energy absorption efficiency (Figure 8), whereby energy absorption efficiency (η) is defined as the ratio of specific energy to the current stress using Equation (1) [22,31,32].

(1) $$\eta =\frac{\int_{0}^{\varepsilon }\sigma \left(\varepsilon \right)d\varepsilon }{\sigma \left(\varepsilon \right)}$$

### 3.3. Mechanical Properties

As the main loading component of LNG cargo containment system is the compressive load (sloshing load and self-gravity) applied to the rise direction of the polymeric foam, five compression tests were conducted in each case to obtain a reliable stress–strain relationship. The average value of the three nearest data in five experiments was used as a representative value for each scenario.

**Figure 9:**
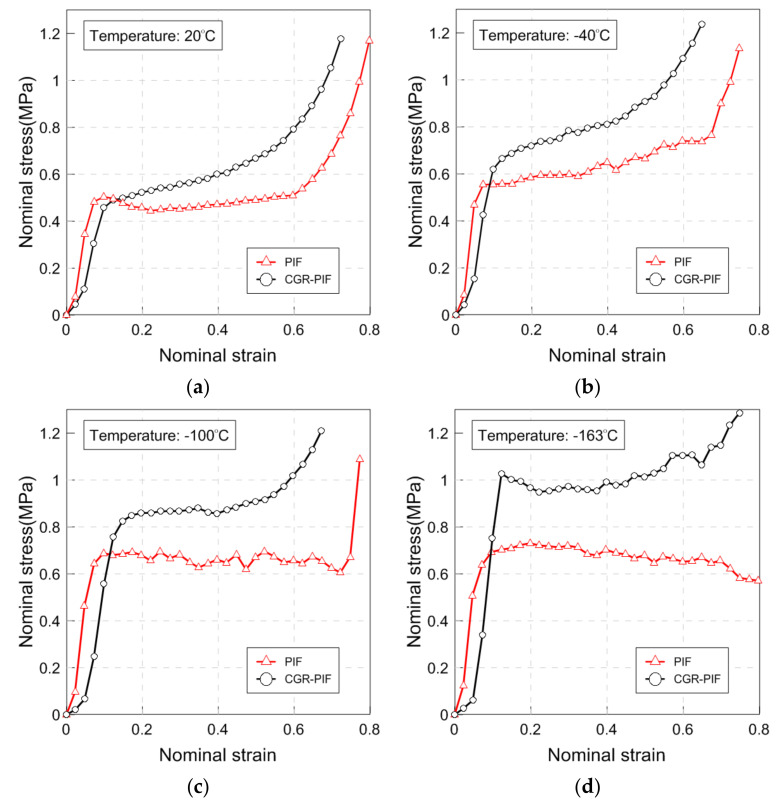
Stress–strain curve of the PIF and CGR-PIF at (**a**) 20, (**b**) −40, (**c**) −100, and (**d**) −163 °C.

Figure 9 shows the stress–strain curve obtained at each temperature (20, −40, −100, and −163 °C). On comparing the effect of the temperature on the compressive strength of each foam, the compressive strength that represents yield stress increased as the temperature decreased, which matches the results of previous research [8,10,16,33]. It was also observed that a lowering in the temperature caused an increase in the stress fluctuation. This is because the cell collapsed at low temperatures exhibiting brittle characteristics, and was generally more pronounced as the temperature decreases [28]. However, in the case of PIF, in contrast to CGR-PIF, at −163 °C, the stress fluctuation disappeared and the stress decreased slightly. It was considered that PIF was friable owing to the occurrence of brittleness fracture at −163 °C, and the plateau region continued up to a strain of 0.8. As can be observed from the stress–strain curve, the compressive strength of PIF and CGR-PIF did not differ greatly at room temperature, and PIF exhibited a decrease in stress after yielding. This is a common phenomenon that occurs in the compression behavior of polymer foams and is known to occur owing to the buckling of the overall cells [34,35]. The difference in compressive strength between PIF and CGR-PIF was found to be larger as the temperature decreased. This is because the chopped glass fibers formed with PIF become stiffer as the temperature decreases [36,37]. In the case of CGR-PIF, strain-hardening at 20 and −40 °C was observed in which the stress increased even after the occurrence of yielding. It was considered that the stress resistance to deformation was increased as the glass fiber had a higher strength than PIF after the occurrence of yielding.

**Figure 10:**
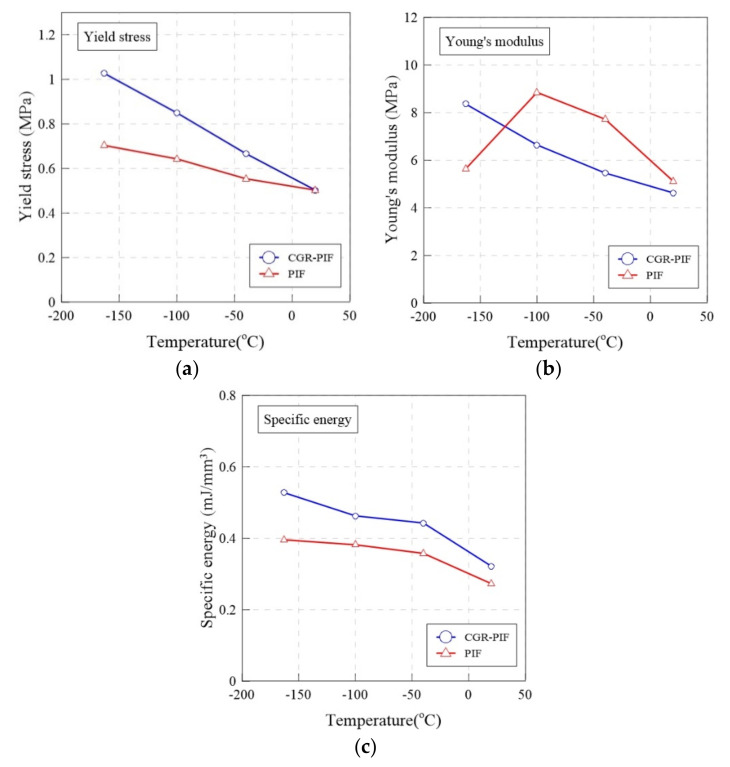
Compressive properties: (**a**) yield stress, (**b**) young’s modulus and (**c**) specific energy of PIF and CGR-PIF at various temperatures.

Figure 10 shows the yield stress, Young’s modulus, and specific energy of the PIF and CGR-PIF in a bar graph. Table 6 shows the compressive properties of the PIF and CGR-PIF at various temperatures (20, −40, −100, and −163 °C). In a quantitative comparison, the compressive strength increased by −10%, 17%, 24%, and 32% as compared to that of neat PIF when the temperatures in the compressive tests were 20, −40, −100, and −163 °C, respectively. At room temperature, the glass fibers in the CGR-PIF did not affect the yield stress owing to the buckling caused by the compressive load. However, the failure strain of the glass fibers in CGR-PIF was greater than that at a low temperature, and it could be analyzed to have a specific energy greater than that of PIF [38,39]. Furthermore, the rate of increase in yield stress increased as the temperature decreased. Considering that the density of the foam increased by 25% owing to the addition of chopped glass fibers, the performance of CGR-PIF at −163 °C was the best. In terms of Young’s modulus, it increased by −10%, −30%, −25%, and 45% as compared to that of neat PIF when the temperatures of the compressive test were 20, −40, −100, and −163 °C, respectively. In general, except for the case of −163 °C, it can be confirmed that the Young’s modulus of PIF was greater than that of CGR-PIF. This is because PIF had a relatively uniform cell structure than CGR-PIF, and thus, it was thought to be resistant to deformation in the elastic region. As mentioned earlier, it can be observed that the Young’s modulus of PIF at −163 °C was lower than that at −100 °C owing to its brittle friability, and the specific energy was calculated to quantitatively analyze its characteristics beyond the elastic region. The area up to the $\varepsilon_{D}$ at which the densification period starts was calculated. Thus, it was confirmed that the specific energy increased as the temperature of both PIF and CGR-PIF decreased. The specific energy of CGR-PIF increased by 17%, 23%, 21%, and 33% as compared with that of neat PIF when the temperatures of the compressive test were 20, −40, −100, and −163 °C, respectively.

### 3.4. Failure Characteristics

**Figure 11:**
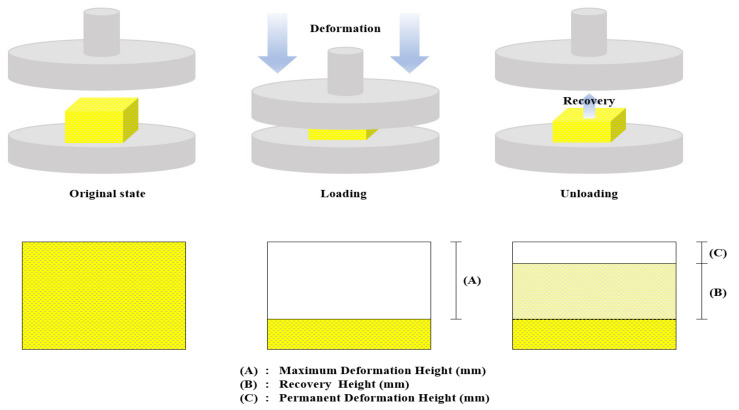
Definition of maximum deformation height and recovery height.

After the polymeric foam is compressively deformed, recovery occurs in the direction in which the deformation occurs. This can cause dimensional instability under LNG loading (compressively deformed) and unloading states (recovery) in LNG insulation system owing to a permanent deformation resulting in strength reduction due to a gap in the insulation structure. In this study, when the load of the universal testing machine applied to the testing sample reached 5 kN, the compressive loading was terminated. As soon as the test was terminated, the maximum deformation height of the tested sample was measured to calculate the maximum deformation ratio. After 24 h at ambient temperature with no external loading, the permanent deformation height of the tested sample was measured to calculate recovery ratio. Figure 11 illustrates the definition of the maximum deformation height and recovery height.

**Figure 12:**
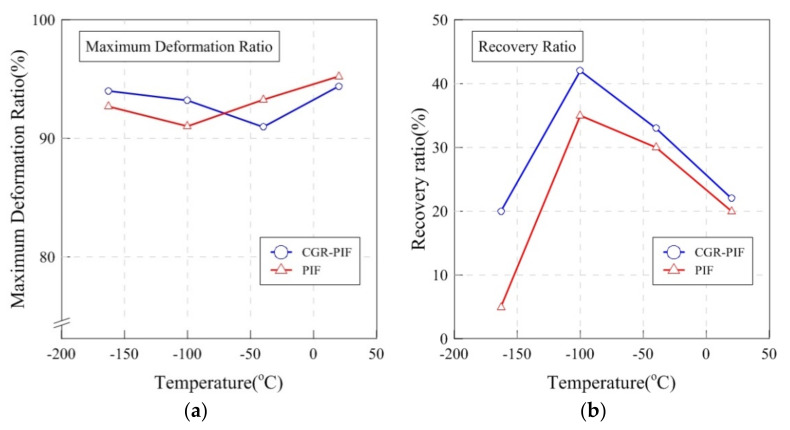
(**a**) Maximum deformation ratio and (**b**) recovery ratio for PIF and CGR-PIF.

**Figure 13:**
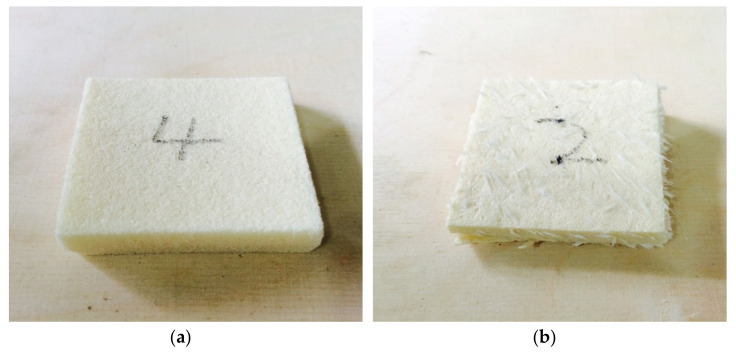

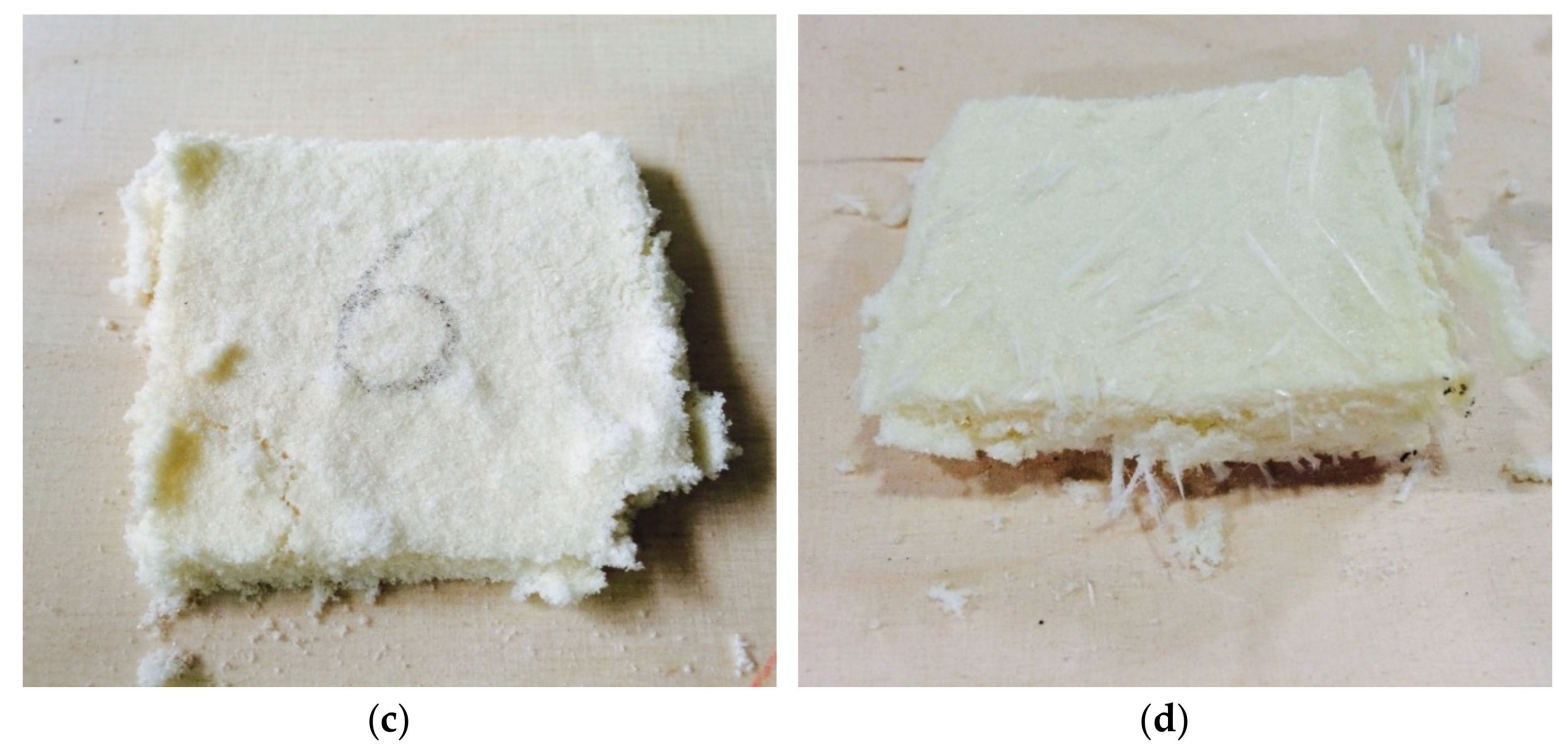
Photograph of permanently deformed specimen after compressive test for (**a**) PIF at 20 °C, (**b**) CGR-PIF at 20 °C, (**c**) PIF at −163 °C, and (**d**) CGR-PIF at −163 °C.

Figure 12 shows a comparison of the maximum deformation ratio and recovery ratio. The maximum deformation ratio was calculated by dividing the maximum deformation height by the height of the sample before the compressive test. The recovery ratio was calculated by dividing the recovery height by the height of the sample before the compression test. As shown in Figure 12a, the maximum deformation ratio for both PIF and CGR-PIF ranged from 90–95%. Generally, no large difference in the maximum deformation ratio was observed as the temperature was varied. Figure 12b shows the recovery ratio of PIF and CGR-PIF with the temperature change. The results of the experiment show that CGR-PIF had a higher recovery ratio than PIF. In addition, the difference in recovery ratio increased as the temperature decreased to −163 °C. This suggests that the glass fibers with a high modulus of elasticity helped the cells to prevent the occurrence of brittle fracture owing to the compressive load [8]. In the case of the trend of the recovery ratio according to the change in temperature, the recovery ratio was found to increase up to −100 °C and then decrease at −163 °C. Generally, when the test was performed at a low temperature, the foam gas condensed in the cell of the polymeric foam. If the test specimen was left at room temperature after the test, the recovery ratio of the foam increased owing to the expansion of the foam gas. However, at −163 °C, the recovery ratio decreased because the cells were brittle crushed, and the cells in the polymeric foam lost their original shape. Figure 13 shows a photograph of the permanently deformed PIF and CGR-PIF specimens after the compressive test at 20 and −163 °C. At −163 °C, the PIF was in a friable state, and it was hardly restored. In contrast, CGR-PIF showed a relatively high recovery of 20% and remained relatively intact with only a slight deformation. As can be observed from the evaluation of the compressive strength, it was considered that when the temperature was lower than −100 °C in the case of PIF, the material was broken, and the compressive strength did not increase any more.

**Figure 14:**
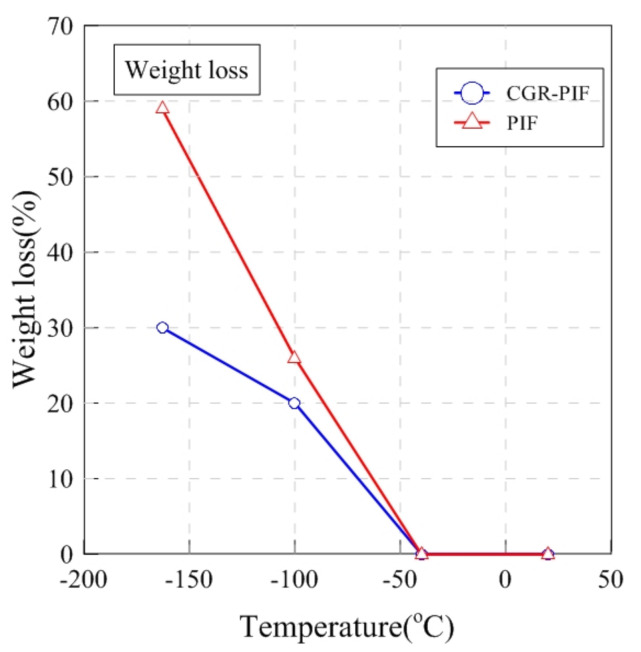
Weight loss ratio for PIF and CGR-PIF.

Brittle fracture occurs when the compressive load is applied to the polymeric foam at a cryogenic temperature. The brittle fracture destroys the side of the polymeric foam specimen such that the degree of brittle fracture can be measured by evaluating the weight loss after the compressive test. Figure 14 shows the weight loss ratio for PIF and CGR-PIF. The weight loss ratio was calculated by dividing the weight obtained after the compressive test by the original weight. Little change was observed in the weight at 20 °C and that at −40 °C. From −100 °C, brittle fracture began to occur in both the materials. At −163 °C, the weight loss of PIF was two times higher than that of CGR-PIF, which shows that the addition of glass fibers helped to prevent the occurrence of brittle fracture.

### 3.5. Thermal Conductivity

**Figure 15:**
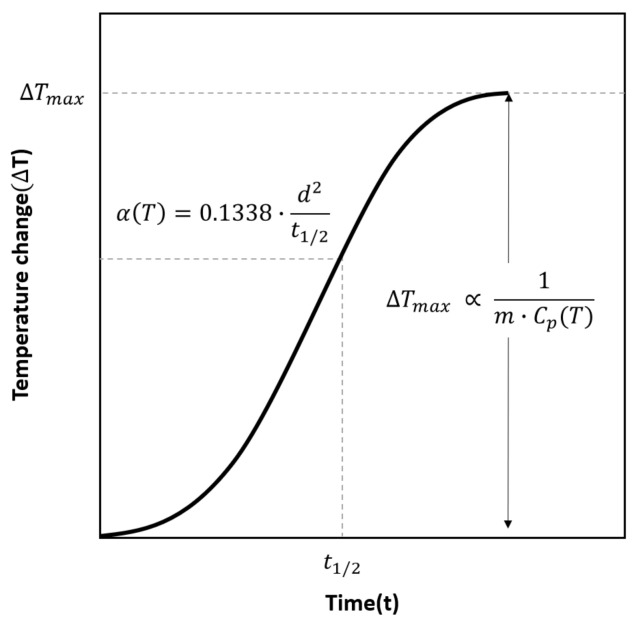
Typical curve of temperature change on the rear surface of the specimen measured by IR detector.

Thermal conductivity is an important characteristic of a thermal insulation material [13]. The heat transfer of PIF primarily occurs through direct conduction, and the thermal conductivity is affected by density, cell distribution, and added materials [10,11,40]. In order to investigate the effect of chopped glass fibers in PIF, its thermal properties were evaluated at an ambient temperature using laser flash analysis. Figure 15 shows typical curve of temperature change measured by IR detector. The thermal diffusivity, $\alpha \left(T\right)$, can be calculated from the measured temperature change on the rear surface of the specimen using the Equation (2), where d is the thickness of the specimen, and $t_{1/2}$ is half of the final rise time value. Furthermore, the specific heat, $C_{p}\left(T\right)$, can be determined from Equation (3), where the maximum value of temperature change, $\Delta T_{max}$, correlates with the known reference specific heat, $C_{p}{\left(T\right)}_{ref}$, which is in LFA-467 analysis program. Then, the specific heat of the test specimen can be calculated through Equation (4).

The test was performed five times for each specimen, and Table 7 shows the average value of the density, thermal diffusivity, specific heat, and thermal conductivity. The thermal conductivity $\lambda \left(T\right)$ of a sample was derived from Equation (5), where $\rho \left(T\right)$ is the apparent density of the sample, and $C_{p}\left(T\right)$ was derived from Equation (4). As a result, it was confirmed that the PIF had a 19% higher thermal diffusivity than CGR-PIF, while CGR-PIF had a 30% higher specific heat than PIF. Therefore, it was confirmed that the PIF and CGR-PIF had a thermal conductivity of 0.0268 W/m·K and 0.0352 W/m·K, respectively. A relatively higher thermal conductivity was observed for CGR-PIF as compared to PIF owing to the addition of chopped glass fibers resulting in smaller cell size, which increased the conduction heat transfer and reduced the trapped gas due to the dense cell structure [26,41,42].

(2) $$\alpha \left(T\right)=0.1338\cdot \frac{d^{2}}{t_{1/2}}$$

(3) $$\Delta T_{max}\;\propto \;\frac{1}{m\cdot C_{p}\left(T\right)}$$

(4) $$C_{p}\left(T\right)=\frac{\Delta T_{max,ref}\cdot m_{ref}}{\Delta T_{max}\cdot m}\cdot C_{p}{\left(T\right)}_{ref}$$

(5) $$\lambda \left(T\right)=\alpha \left(T\right)\cdot C_{p}\left(T\right)\cdot \rho \left(T\right)$$

## 4. Concluding Remarks

In this study, CGR-PIF was synthesized based on the amount of glass fiber that is generally added to commercially available R-PUF to investigate the effect of chopped glass fibers on the mechanical and thermal performance of PIF. The results obtained in this study are summarized below.

- In terms of a comparison of the shapes of the cells of the synthesized CGR-PIF and PIF, the cells of the PIF were found to be relatively uniform in shape, and the cells of the closed form were completely present. However, in the case of CGR-PIF, it is observed that the cell growth around the glass fiber was hindered by the glass fiber, and consequently, the cell size around the chopped glass fiber was relatively small.
- In terms of mechanical behavior of PIF and CGR-PIF, the difference in compressive strength increases as the temperature decreases. Considering that the density is the important parameter when the volume of LNG cargo containment system (CCS) is fixed, the mechanical performance of CGR-PIF at −163 °C is the most superior in terms of compressive strength, Young’s modulus, and specific energy. In fact, when a large load is applied to the PIF used in an LNG fuel CCS, it is better to use CGR-PIF without increasing the thickness of the PIF in terms of mechanical performance.
- In terms of failure characteristics, generally, the recovery ratio of CGR-PIF was higher than that of PIF. Especially at −163 °C, which is the temperature at which LNG is stored, it was confirmed that CGR-PIF recovered 20% whereas PIF recovered only slightly. Furthermore, in the evaluation of the degree of brittle fracture, the weight loss of PIF is found to be two times higher than that of CGR-PIF. Chopped glass fibers can compensate for the disadvantage of PIF becoming friable at −163 °C.
- In terms of thermal conductivity, it was confirmed that the insulation performance of PIF was lowered owing to the addition of chopped glass fibers.

## Figures and Tables

**Table 1:** Properties of polyol mixture used for the production of PIFs.

Property	Unit
Hydroxyl number	238 mg KOH/g
Density	1.23 g/cm^3^
Viscosity	3300 mPa·s
Water content	0.1 wt.%

**Table 2:** Material characteristics of chopped glass fibers.

Characteristics	Unit
Thermal Expansion Coefficient	9 × 10^−6^ /K
Density	2.8 × 10^−3^ kg/m^3^
Tensile Strength	1.5 GN/m^2^
Young’s modulus	74 GN/m^2^
Strain to failure	2%
Diameter	10 μm

**Table 3:** Composition and mixing conditions of the PIF and CGR-PIF.

Materials	PIF	CGR-PIF
Polyol mixture	1000 g	1000 g
Polymeric M50	1800 g	1800 g
Glass fiber	-	500 g
Mixing RPM	6000 rpm	6000 rpm
Mixing time	15 s	15 s
Mixing temperature	23 °C	23 °C
Gel time(s)	150 s	150 s
Density	59 kg/m^3^	75 kg/m^3^

**Table 4:** Conditions for the cryogenic compressive tests.

Material	Glass Fiber (wt.%)	Temperature (°C)	Specimen(EA)
PIF	0	−20	5
		−40	5
		−100	5
		−163	5
CGR-PIF	15	−20	5
		−40	5
		−100	5
		−163	5

**Table 5:** Average cell size and maximum cell size of PIF and CGR-PIF.

Material	Directions	Ave. Cell Size (μm)	Max. Cell Size (μm)	Standard Deviation (μm)
PIF	Perpendicular to foaming direction	366	510	81
	Parallel to foaming direction	335	600	131
CGR-PIF	Perpendicular to foaming direction	283	420	79
	Parallel to foaming direction	241	350	57

**Table 6:** Compressive properties of PIF and CGR-PIF at various temperatures.

Material	Temperature (°C)	Young’s Modulus (MPa)	Yield Stress (MPa)	Specific Energy (mJ/mm^3^)
PIF	20	5.126	0.503	0.273
	−40	7.727	0.554	0.358
	−100	8.852	0.643	0.382
	−163	5.735	0.703	0.396
CGR-PIF	20	4.632	0.457	0.322
	−40	5.466	0.666	0.443
	−100	6.658	0.850	0.463
	−163	8.368	1.027	0.528

**Table 7:** Average thermal properties of PIF and CGR-PIF at 20 °C.

Material	Density (g/cm^3^)	Diffusivity (mm^2^/s)	Specific Heat (J/g/K)	Thermal Conductivity (W/m·K)
PIF	0.0586	0.4246	1.077	0.0268
CGR-PIF	0.0757	0.3572	1.3004	0.0352

## Data Availability

The data presented in this study are available on request from corresponding author.

## References

[1] Ruehl C., Giljum J. (2011). BP Energy Outlook 2030.

[2] Kumar S., Kwon H.T., Choi K.H., Hyun Cho J., Lim W., Moon I. (2011). Current status and future projections of LNG demand and supplies: A global prospective. Energy Policy.

[3] Kanbur B.B., Xiang L., Dubey S., Choo F.H., Duan F. (2017). Cold utilization systems of LNG: A review. Renew. Sustain. Energy Rev..

[4] Querol E., Gonzalez-Regueral B., García-Torrent J., García-Martínez M.J. (2010). Boil off gas (BOG) management in Spanish liquid natural gas (LNG) terminals. Appl. Energy.

[5] Malenica S., Diebold L., Kwon S.H., Cho D.S. (2017). Sloshing assessment of the LNG floating units with membrane type containment system where we are?. Mar. Struct..

[6] Thirumal M., Khastgir D., Singha N.K., Manjunath B.S., Naik Y.P. (2008). Effect of foam density on the properties of water blown rigid polyurethane foam. J. Appl. Polym. Sci..

[7] Chun M.S., Kim M.H., Kim W.S., Kim S.H., Lee J.M. (2009). Experimental investigation on the impact behavior of membrane-type LNG carrier insulation system. J. Loss Prev. Process Ind..

[8] Park S.B., Lee C.S., Choi S.W., Kim J.H., Bang C.S., Lee J.M. (2016). Polymeric foams for cryogenic temperature application: Temperature range for non-recovery and brittle-fracture of microstructure. Compos. Struct..

[9] Berardi U. (2017). The impact of temperature dependency of the building insulation thermal conductivity in the Canadian climate. Energy Procedia.

[10] Kim J.M., Kim J.H., Ahn J.H., Kim J.D., Park S., Park K.H., Lee J.M. (2018). Synthesis of nanoparticle-enhanced polyurethane foams and evaluation of mechanical characteristics. Compos. Part B Eng..

[11] Ulrich H. (1981). Recent Advances in Isocyanurate Technology. J. Cell. Plast..

[12] Panowicz R., Miedzińska D. (2012). Numerical and experimental research on polyisocyanurate foam. Comput. Mater. Sci..

[13] Goods S.H., Neuschwanger C.L., Henderson C.C., Skala D.M. (1998). Mechanical properties of CRETE, a polyurethane foam. J. Appl. Polym. Sci..

[14] Zhao C., Yan Y., Hu Z., Li L., Fan X. (2015). Preparation and characterization of granular silica aerogel/polyisocyanurate rigid foam composites. Constr. Build. Mater..

[15] Tran V.H., Kim J.D., Kim J.H., Kim S.K., Lee J.M. (2020). Influence of Cellulose Nanocrystal on the Cryogenic Mechanical Behavior and Thermal Conductivity of Polyurethane Composite. J. Polym. Environ..

[16] Han D.S., Park I.B., Kim M.H., Noh B.J., Kim W.S., Lee J.M. (2010). The effects of glass fiber reinforcement on the mechanical behavior of polyurethane foam. J. Mech. Sci. Technol..

[17] Siegmann A., Kenig S., Alperstein D., Narkis M. (1983). Mechanical behavior of reinforced polyurethane foams. Polym. Compos..

[18] Şerban D.A., Weissenborn O., Geller S., Marşavina L., Gude M. (2016). Evaluation of the mechanical and morphological properties of long fibre reinforced polyurethane rigid foams. Polym. Test..

[19] Gluck D.G., Hagan J.R., Hipchen D.E. (1980). Glass Fiber Reinforced Isocyanurate-Urethane Foams. J. Cell. Plast..

[20] Zhao Y.H., Wu Z.K., Bai S.L. (2016). Thermal resistance measurement of 3D graphene foam/polymer composite by laser flash analysis. Int. J. Heat Mass Transf..

[21] Dawson J.R., Shortall J.B. (1982). The microstructure of rigid polyurethane foams. J. Mater. Sci..

[22] Gibson L.J., Ashby M.F. (2014). Cellular Solids: Structure and Properties.

[23] Kim S.H., Park H.C., Jeong H.M., Kim B.K. (2010). Glass fiber reinforced rigid polyurethane foams. J. Mater. Sci..

[24] Jung H.C., Ryu S.C., Kim W.N., Lee Y.B., Choe K.H., Kim S.B. (2001). Properties of rigid polyurethane foams blown by HCFC 141B and distilled water. J. Appl. Polym. Sci..

[25] Chuang Y.C., Li T.T., Huang C.H., Huang C.L., Lou C.W., Chen Y.S., Lin J.H. (2016). Protective rigid fiber-reinforced polyurethane foam composite boards: Sound absorption, drop-weight impact and mechanical properties. Fibers Polym..

[26] Barczewski M., Kurańska M., Sałasińska K., Michałowski S., Prociak A., Uram K., Lewandowski K. (2020). Rigid polyurethane foams modified with thermoset polyester-glass fiber composite waste. Polym. Test..

[27] Mane J.V., Chandra S., Sharma S., Ali H., Chavan V.M., Manjunath B.S., Patel R.J. (2017). Mechanical Property Evaluation of Polyurethane Foam under Quasi-static and Dynamic Strain Rates- An Experimental Study. Procedia Eng..

[28] Saha M.C., Mahfuz H., Chakravarty U.K., Uddin M., Kabir M.E., Jeelani S. (2005). Effect of density, microstructure, and strain rate on compression behavior of polymeric foams. Mater. Sci. Eng. A.

[29] Tu Z.H., Shim V.P.W., Lim C.T. (2001). Plastic deformation modes in rigid polyurethane foam under static loading. Int. J. Solids Struct..

[30] Subhash G., Liu Q., Gao X.L. (2006). Quasistatic and high strain rate uniaxial compressive response of polymeric structural foams. Int. J. Impact Eng..

[31] Avalle M., Belingardi G., Montanini R. (2001). Characterization of polymeric structural foams under compressive impact loading by means of energy-absorption diagram. Int. J. Impact Eng..

[32] Li P., Guo Y.B., Zhou M.W., Shim V.P.W. (2019). Response of anisotropic polyurethane foam to compression at different loading angles and strain rates. Int. J. Impact Eng..

[33] Park S.B., Choi S.W., Kim J.H., Bang C.S., Lee J.M. (2016). Effect of the blowing agent on the low-temperature mechanical properties of CO2- and HFC-245fa-blown glass-fiber-reinforced polyurethane foams. Compos. Part B Eng..

[34] Lee J.H., Kim S.K., Park S., Park K.H., Lee J.M. (2018). Unified constitutive model with consideration for effects of porosity and its application to polyurethane foam. Compos. Part B Eng..

[35] Goods S.H., Neuschwanger C.L., Whinnery L.L., Nix W.D. (1999). Mechanical properties of a particle-strengthened polyurethane foam. J. Appl. Polym. Sci..

[36] Yu Y.H., Choi I., Nam S., Lee D.G. (2014). Cryogenic characteristics of chopped glass fiber reinforced polyurethane foam. Compos. Struct..

[37] Shen H., Nutt S. (2003). Mechanical characterization of short fiber reinforced phenolic foam. Compos. Part A Appl. Sci. Manuf..

[38] Mehdikhani M., Gorbatikh L., Verpoest I., Lomov S.V. (2019). Voids in fiber-reinforced polymer composites: A review on their formation, characteristics, and effects on mechanical performance. J. Compos. Mater..

[39] Dutta P.K. (1994). Low-Temperature Compressive Strength of Glass-Fiber-Reinforced Polymer Composites. J. Offshore Mech. Arct. Eng..

[40] Choi S.W., Roh J.U., Kim M.S., Lee W.I. (2012). Analysis of two main LNG CCS (cargo containment system) insulation boxes for leakage safety using experimentally defined thermal properties. Appl. Ocean Res..

[41] Hejna A., Kosmela P., Kirpluks M., Cabulis U., Klein M., Haponiuk J., Piszczyk Ł. (2018). Structure, Mechanical, Thermal and Fire Behavior Assessments of Environmentally Friendly Crude Glycerol-Based Rigid Polyisocyanurate Foams. J. Polym. Environ..

[42] Mosiewicki M.A., Dell’Arciprete G.A., Aranguren M.I., Marcovich N.E. (2009). Polyurethane foams obtained from castor oil-based polyol and filled with wood flour. J. Compos. Mater..

